# A Vision-Based Automated Guided Vehicle System with Marker Recognition for Indoor Use

**DOI:** 10.3390/s130810052

**Published:** 2013-08-07

**Authors:** Jeisung Lee, Chang-Ho Hyun, Mignon Park

**Affiliations:** 1 School of Electrical and Electronic Engineering, Yonsei University, 134 Shinchon-Dong, Seodaemun-Gu, Seoul 120-749, Korea; E-Mails: leejaisung@yonsei.ac.kr (J.L.); mignpark@yonsei.ac.kr (M.P.); 2 Division of Electrical Electronic and Control Engineering, Kongju National University, Cheonan, Chungnam 331-717, Korea

**Keywords:** marker recognition, automated guided vehicle, vision based AGV system, bird's eye view, marker matching

## Abstract

We propose an intelligent vision-based Automated Guided Vehicle (AGV) system using fiduciary markers. In this paper, we explore a low-cost, efficient vehicle guiding method using a consumer grade web camera and fiduciary markers. In the proposed method, the system uses fiduciary markers with a capital letter or triangle indicating direction in it. The markers are very easy to produce, manipulate, and maintain. The marker information is used to guide a vehicle. We use hue and saturation values in the image to extract marker candidates. When the known size fiduciary marker is detected by using a bird's eye view and Hough transform, the positional relation between the marker and the vehicle can be calculated. To recognize the character in the marker, a distance transform is used. The probability of feature matching was calculated by using a distance transform, and a feature having high probability is selected as a captured marker. Four directional signals and 10 alphabet features are defined and used as markers. A 98.87% recognition rate was achieved in the testing phase. The experimental results with the fiduciary marker show that the proposed method is a solution for an indoor AGV system.

## 1. Introduction

An Automated Guided Vehicle (AGV) is an intelligent mobile robot widely used to move objects or perform tasks in various places such as the industrial field, harbors, warehouses or dangerous working areas where human could not work in. Self-navigation is a key technology of the AGV system. GPS-based navigation systems are widely used in positioning systems, and GPS is a good solution for outdoor navigation, but GPS may not be available in indoor environments, because the satellites' signals are blocked inside buildings [1]. Various types of indoor localization methods using ultrasonic, infrared, magnetic, and radio sensors have been proposed [2–4]. An indoor positioning system with these sensors requires infrastructure to transmit the signal and robots that are equipped with a receiver and amplifier. Deploying transmitters to create an infrastructure is costly.

In order to reduce the cost and obtain robust positioning results, vision sensors have been applied to the AGV system. Jung *et al.* [5] used edge detection, square angular estimation and a Hough transform to estimate lanes on a road. Wang *et al.* [6] applied a B-snake spline as a geometric model to represent the road. Zhou *et al.* [7] also used a geometrical model and Gabor filter to detect a lane. Some other researchers [8,9] have proposed an indoors lane-following system. They showed that algorithms could be effective indoors.

However, the algorithms did not take location information into account. In an industrial environment, it is very important to know the exact location of the AGV. Especially if there are many robots cooperating in the same space, each location must be checked to avoid bottlenecks. Yu *et al.* [10] tried to detect some numbers beside the guide lane to check the location of the AGV. They used a neural network algorithm to recognize the numbers. Sabican *et al.* [11] also used marker recognition to inform the robot of where to go in the intersection.

In some other studies [12–14], they used a fisheye lens with some markers on the ceiling to obtain the exact location of the AGV instead of using a lane tracking method, because installing a lane is costly and changing a route is sometimes difficult. The method of using markers on the ceiling with a fisheye lens can be very useful in indoors scenarios, but this method is not appropriate for general industrial environments that have high ceilings or wide spaces. Petriu *et al.* [15] proposed a method of recognizing bit codes on the floor. The floor is permanently encoded with the terms of a pseudo random binary array. By using the binary codes, the AGV can move in any direction on the floor. Kim *et al.* [16] also used a marker recognition method to locate the AGV without guideline. They applied Artificial Neural Networks (ANNs) to recognize letters of the alphabet on the marker. Their main concerns were reducing the cost of the installation, maintaining the system and the usability. ARToolkit is widely used for marker recognition in various fields and it can also be used to AGV system with marker, but it is week in occlusion problems and illumination changes.

In this paper, we have developed a vision-based AGV system using fiduciary markers. If there is no guideline on the floor, the mobile robot needs to find its location by using only markers. In Section 2, we show a method of estimating the location of the robot using a maker. If a marker is detected by a camera, we can obtain a great deal of information from the marker; including the distance from the robot, the location and the rotation angle of the marker. The HSV color space is used to estimate candidates of the marker. We use only hue and saturation values to reduce the influence of sudden illumination changes.

The bird's eyes view image and Hough transform method were applied to robustly extract a marker on an image and to estimate the position of the marker. Jung *et al.* [17] also used the Hough transform to recognize the marker. We modified their approach to obtain a more robust recognition result even if there are some occlusion problems and to determine the rotation angle of the marker at the same time. To recognize the character in the marker, a distance transform is used. The probability of feature matching can be calculated by using a distance transform, and a feature having high probability is selected as a captured marker. Four directional signals and 10 alphabet features are defined and used as markers. In Section 3, we show the performance of each algorithm and the effectiveness of the developed method.

## 2. Method of Marker Recognition

**Figure 1.**
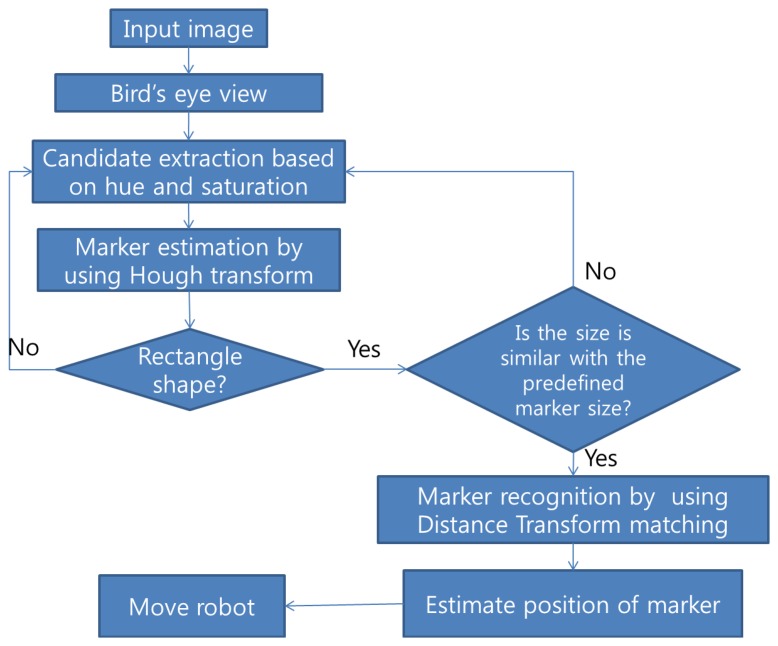
Marker detection and robot control process.

The developed algorithm consists of five steps: image change to bird's eye view, candidate extraction, marker estimation by using Hough transform, marker matching, and robot control. A flowchart of the method is shown in Figure 1. There are several advantages in using the bird's eye view image. By using the bird's eye view, we can obtain a more robust result during marker recognition and more accurate information about the situation of the marker such as the distance between the marker and robot, the rotation angle of the marker, and the direction of the marker from the robot. If we use bird's eye view, the markers should have a rectangular shape. This feature can reduce the complexity of the marker recognition process. In the candidate extraction step, we used the HSV color space with a specific color marker. Markers generally have been used with simple black and white colors. Adaptive threshold in gray scale imaging has been used to extract the marker candidates. However, if we use specific color information instead of using an adaptive threshold, we can significantly reduce the number of candidates and computational cost. We neglect illumination information and use only hue and saturation values to extract candidates, because illumination can change significantly according to environment. After extracting candidates, we applied a Hough transform to detect a marker and to estimate the rotation angle of the marker. The Hough transform can compensate for partial marker errors such as occlusion problems. To recognize a sign in the marker, a distance transform was used. Then, the position of the marker such as distance and direction from a robot was calculated. We used the obtained marker information to control the robot.

### 2.1. Experiment Environments

**Figure 2.**
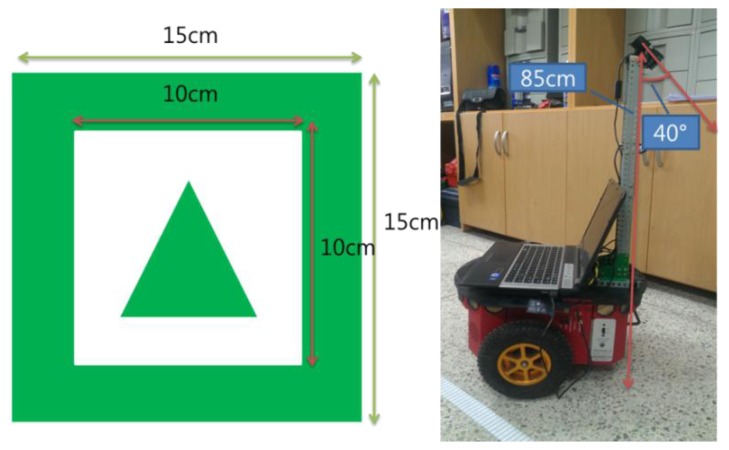
Marker size and robot with installed cameras.

We used a Pioneer3DX robot and a Microsoft webcam. The webcam is very inexpensive, but it is adequate for the developed algorithm. Figure 2 shows a robot with the installed camera. We installed a camera in a high place to reduce the influence of diffused reflection and to increase the range of vision. The camera is about 85 cm high and its down angle is 40°. The marker recognition range is from 60 to 240 cm. We used the center of the robot wheels as the starting point for the distance between the robot and markers, because using the center of the robot wheels as the starting point is reasonable to precisely control the robot.

**Figure 3.**
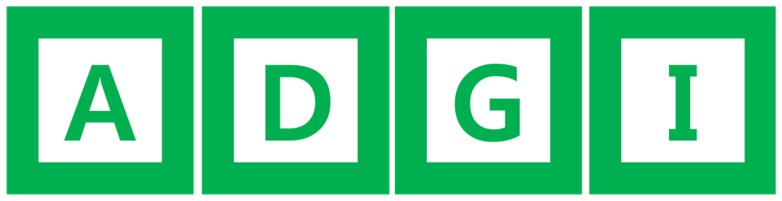
Fiduciary Markers with alphabet ‘A’, ‘D’, ‘G’, and ‘I’.

Figure 2 also shows an example of a marker. Markers are composed of three sectors: the outer green border, the inner white rectangle, and a sign such as a direction sign or letters of the alphabet. In the figure shown, the marker has a triangle sign. This sign indicates the direction for moving forward. In the proposed method, since we can obtain a robust angle of marker rotation, we can provide four kinds of instructions by using only one direction sign marker to go straight, turn left, turn right, stop, *etc.* The marker used in the experiment is 15 cm wide and 15 cm long and has a simple triangle figure in the center of the marker. There is no particular reason that we choose the triangle shape to indicate direction. Any other kind of shape can be used. Figure 3 shows examples of markers with letters of the alphabet. Each letter sign can be used to indicate a special place. In this experiment, we used 10 alphabet letters from A to J as marker signs.

Vision systems can be significantly influenced by diffused reflection of light. It is difficult to reduce the influence of light with an algorithm method, so we used a polarizing filter that can effectively reduce the influence of diffused reflection.

### 2.2. Bird's Eye View Image and Distance Estimation

**Figure 4.**
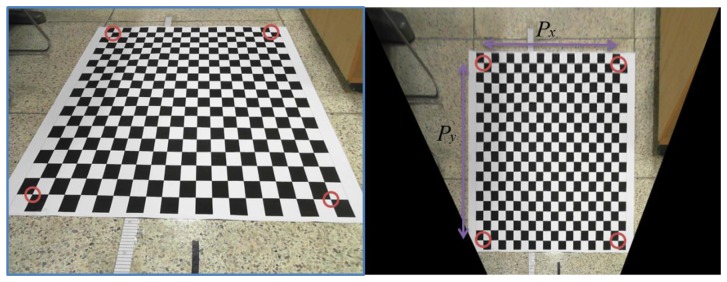
Experiment environment (**left**) and bird's eye view result (**right**).

Figure 4 shows the experiment environment and bird's eye view result. In the figure, there is a checker board that is used for calibration. If we know the actual length of each block on the checker board, we can calculate the real length per pixel by comparing the real distance with the number of pixels between detected corner points. In the bird's eye view image, we defined *P_x_* as the number of pixels between two corner points of the check board in the *x* direction and *P_y_* as the number of pixels between two corner points of the check board in *y* direction. If *d_x_* and *d_y_* are real distances of *P_x_* and *P_y_*, the distances per pixel *r_x_* and *r_y_* can be obtained as follows:

(1) $$\begin{array}{c} r_{x}=d_{x}/P_{x}\\ r_{y}=d_{y}/P_{y} \end{array}$$

**Figure 5.**
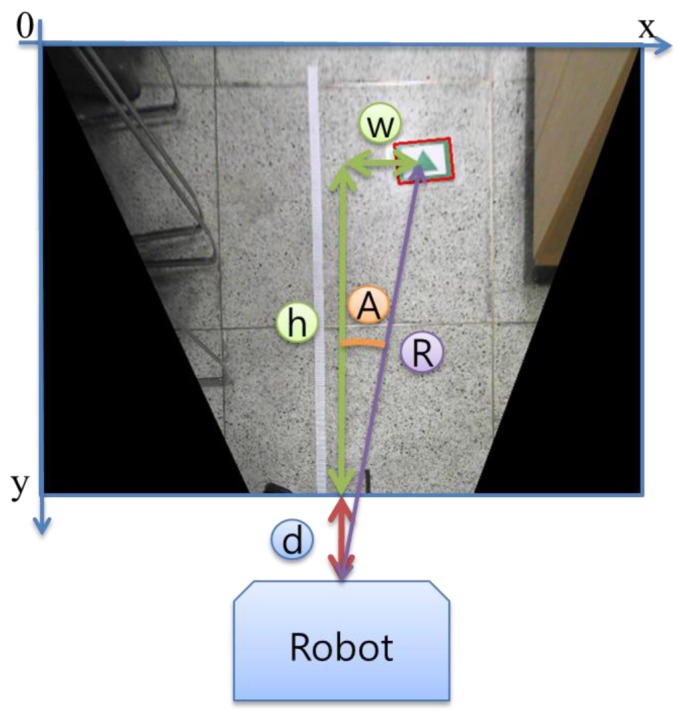
Distance and direction from the robot to the marker.

If a marker is detected as Figure 5, we can calculate the distance between the marker and the robot. The direction of the marker from the robot is also obtained by using Equation (2):

(2) $$\begin{array}{l} R=\sqrt{{\left(d+r_{y}\times h\right)}^{2}+{\left(r_{x}\times w\right)}^{2}}\\ ∠A=\tan^{-1}((r_{x}\times w)/(D+r_{y}\times h)) \end{array}$$

In Figure 5, character *d* refers to the distance between the center of the robot wheels and the closest location in the input image. Character *h* is the number of pixels between two points on the *y*-axis and character w describes the number of pixels between two points on the *x*-axis. In Equation (2), we multiply *r_x_* and *r_y_* by the values to obtain the real distance. Character *A* refers to the direction angle of the marker from the robot and *R* indicates the distance between the robot and the marker. Each interval between image pixels is not continuous, but discrete. Therefore, there could be some errors in the value of the distance per pixel. To reduce the errors, we used as large an image as possible. In this research, we used a 640 × 480 pixel size image.

### 2.3. Marker Candidate Extraction

**Figure 6.**
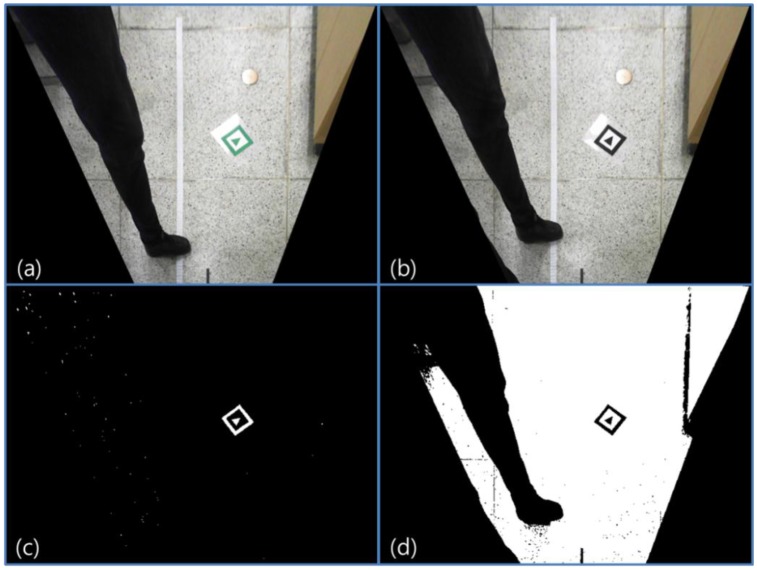
Comparison between binary threshold method in Gray scale image and specific color extraction method with hue and saturation values. (**a**) and (**b**) are input images. (**c**) is the result of extracting a specific color. (**d**) is the result of a binary threshold in the Gray scale image of (**b**).

A specific color marker is used to extract marker candidates in the HSV color space. In many other research projects, black and white color markers have been used with gray scale images. However, if we use a specific color marker, we can more easily extract candidates by using specific ranges of hue and saturation. We do not use illumination values in the HSV color space to extract candidates, because as mentioned, vision systems are easily influenced by illumination changes. Figure 6 compares the binary threshold method with a Gray scale image and the specific color extraction method in HSV color space. Figure 6a,b show the input images. Figure 6c is the result of extracting candidates by using specific range of hue and saturation in HSV image of Figure 6a. Figure 6d is the result of the binary threshold in the Gray scale image of Figure 6b.

In the figure, we can confirm that by using the HSV color space, we can reduce the number of unnecessary candidates. This means that processing costs can also be reduced. Hue and saturation are relatively robust to illumination change compared with RGB or gray color values. After extracting candidates, contour and size of the candidates are calculated. Candidates should have a similar size with predefined markers. Because we use the bird's eye view image, the marker image size is always the same in any place on the image. If the size of a candidate is larger or smaller than the predefined marker size, the candidate is ignored.

### 2.4. Marker Recognition and Angle of Rotation Calculation by Using Hough Transform

**Figure 7.**
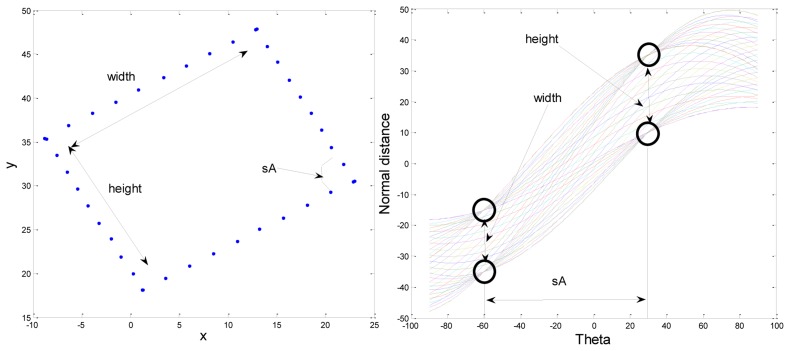
An example of Hough transform of a marker.

The Hough transform method was applied to the extracted candidates for estimating real markers. The marker should have a square shape in the image, because it has a square shape when we use the bird's eye view image. If the contour of the candidate is square, there would be four points with many overlapping Hough lines. By using this character, we can estimate the real marker. Figure 7 shows an example of a Hough transform result of a square shape contour. In the figure, there are four points with many overlapping Hough lines. *sA* refers to the angle difference between the two edges of a square marker. *sA* should be 90°, because the marker has a square shape. The difference between two points in the same angle refers to the height or the width of a square marker.

The purposes of using the Hough transform are estimating the real marker and calculating the rotation angle of the marker. When we applied the Hough transform, we considered the shape of the marker. A general Hough transform has a range from −90 to 90°, but we reduce the range and stack the Hough transform result on from 0 to 89°. If *θ* is less than 0°, the Hough transform value of *θ* + 90° should be added to the value of *θ* degrees because the adjacent edges in the square should have a 90° difference. Also, if *θ* is 90°, the Hough transform value should be added to the value of 0°.

We did not calculate whole Hough transform results, but only compared each two points of the contour to obtain an angle as follows:

(3) $$\theta =\text{round}(\tan^{-1}((x_{1}-x_{2})/(y_{2}-y_{1}))).$$

Each (*x*_1_, *y*_1_) and (*x*_2_, *y*_2_) describe two points among the contours of candidates. The *round* function is used to find the closest integer value. We stacked the *θ* to estimate the marker and to obtain the rotation angle of the marker as follows:

(4) $$\begin{array}{ll} f(\theta \geq 0\;\text{and}\;\theta !=90) & \text{Angtmp}(\theta )=\text{Angtmp}(\theta )+1\\ \text{elseif}\;(\theta <0) & \text{Angtmp}(\theta +90)=\text{Angtmp}(\theta +90)+1\\ \text{elseif}\;(\theta ==90) & \text{Angtmp}(0)=\text{Angtmp}(0)+1 \end{array}$$

**Figure 8.**
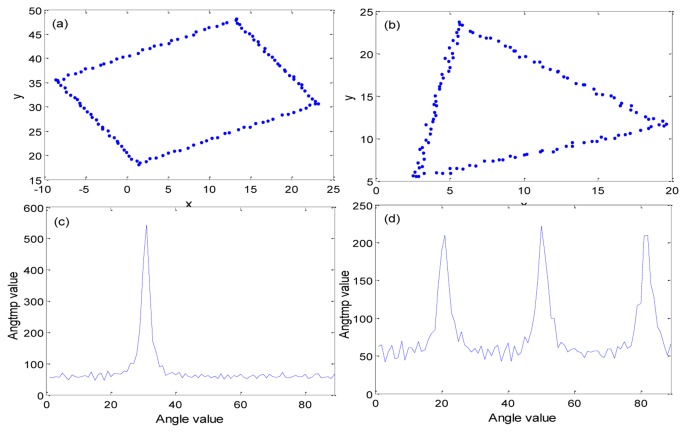
Example of *Angtmp* stack results in Hough transform. (**a**) and (**b**) are input contour points. (**c**) and (**d**) show *Angtmp* stack results.

We used *θ* as an integer value. This means that the resolution in the rotation angle of the marker is also 1°. It is possible to improve the resolution by increasing the range of *θ*. But we think that resolution of one degree is enough, because the mobile robot also has more than one degree of rotation accuracy. In Equation (4), a high value of *Angtmp* implies a high intersection with the Hough transform. If the contour is square, *Angtmp* has one high pick. Otherwise, if the contour is not square, *Angtmp* will have more than one pick. Figure 8 shows an experiment result of *Angtmp*. Figure 8a,b are input contour points. The contour points were made by manually for simple tests.

In a real experiment, the contour points can be obtained by using openCV 2.4 (http://opencv.org) function after extracting candidates. Figure 8a has a square shape contour and Figure 8b has a triangle shape. Figure 8c is the result of a Hough transform for the square shape contour and Figure 8d is the result of Hough transform with the triangle shaped contour. In Figure 8c,d, the *x*-axis angle ranges from 0 to 89° and the *y*-axis refers to the intensity of intersection of the Hough transform. From that result, we can confirm that *Angtmp* has an effect on square shape contour and *Angtmp* has many effects on other shape contours. By using this character, we can find correct markers from candidates. When *Angtmp* has one strong pick, the *x*-axis value of the strong pick indicates the rotation angle of the marker. We will identify the *x*-axis value of the strong pick as the parameter *Angle*. After extracting the marker, four corner points of the marker should be calculated to rotate the image and match it with the predefined marker dataset. If we know the linear equation of the edges, we can obtain four corner points by calculating intersection points.

To obtain edge line equations, we calculate the closest distance between a line passing a contour point and the origin of coordinates. If a line passing a point has a specific slope, there is only one point on the line to determine the shortest distance from the origin of coordinates. If there is a point (*x*,*y*) in two-dimensional coordinates (*X*-axis and *Y*-axis), a line passing the point can be express as follows:

(5) $$\left(Y-y)=\frac{\sin\theta }{\cos\theta }(X-x\right)$$

Because the *y*-axis coordinate is inversed in the image, the equation should be changed as follows:

(6) $$\left(Y-y)=-\frac{\sin\theta }{\cos\theta }(X-x\right)$$

Therefore, the closest distance from the origin to a line can be obtained as follows:

(7) D=|cosθ×y+sinθ×x|

If we change the *θ* to *Angle* and we consider that there is another vertical line, the closest distance can be obtained as follows:

(8) $$\begin{array}{l} \text{Dw}=x\times \cos(\text{Angle})+y\times \sin(\text{Angle})\\ \text{Dh}=x\times \cos(\text{Angle}-\pi /2)+y\times \sin(\text{Angle}-\pi /2) \end{array}$$

**Figure 9.**
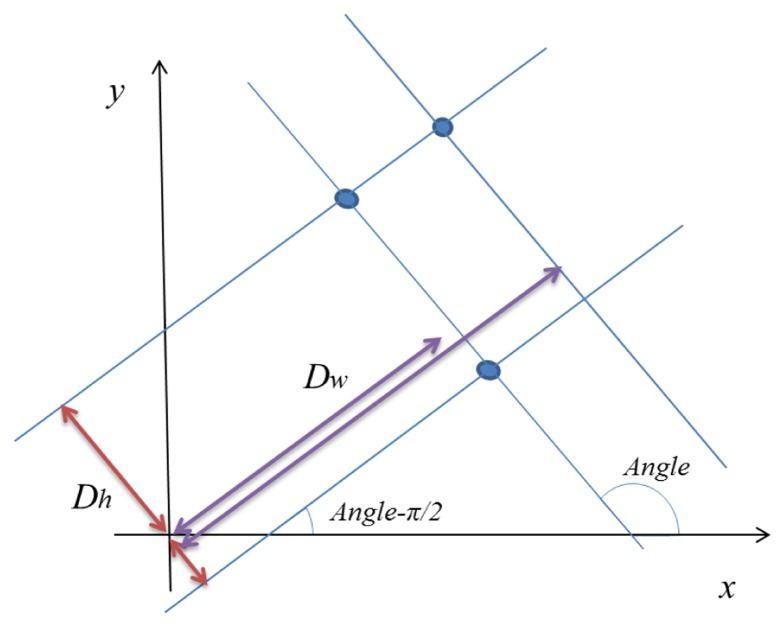
Example of *Dh* and *Dw* with some points.

In the equation, *Dw* and *Dh* refer to the closest distance between the lines passing a contour point (*x*,*y*) and the origin of coordinates. The *Angle* is already obtained and fixed by using the Hough transform, so there are two lines passing a point. One line has the slope *Angle* and *Dw* is the distance from the origin of the coordinate. Another line has the slope (*Angle* – *π*/2) and *Dh* is the distance from the origin of the coordinate. We stacked the *Dw* and *Dh* values at all contour points (*x*,*y*) and find the stochastically high components to select the proper *Dw* and *Dh*. Figure 9 shows an example of *Dh* and *Dw* with some points. The first result of a point is stacked as follows:

(9) $$\begin{array}{cc} \{\begin{array}{l} {\text{Dtmp}}_{i}(n)=D_{i}(j)\\ {\text{cnt}}_{i}(n)=1\\ L=1 \end{array}, & \text{where}\{\begin{array}{l} i=w\;\text{or}\;h\\ n=1,j=1 \end{array} \end{array}$$

Every *j*th new point, we find the minimum difference between *Dtmp_i_*(*n*) and *D_i_*(*j*) and its address *k* in the stack:

(10) $$\begin{array}{cc} {\text{Dist}}_{i}(k)=\underset{\forall n}{\min}({\text{Dtmp}}_{i}(n)-D_{i}(j)), & \text{where}\;k\subset n \end{array}$$

If (*Dist_i_*(*k*) < *th*_*D*), *D_i_*(*j*) belong to *Dtmp_i_*(*k*) and *Dtmp_i_*(*k*) is updated as follows:

(11) $$\{\begin{array}{l} {\text{Dtmp}}_{i}(k)=({\text{cnt}}_{i}(k)\times {\text{Dtmp}}_{i}(k)+D_{i}(j))/({\text{cnt}}_{i}(k)+1)\\ {\text{cnt}}_{i}(k)={\text{cnt}}_{i}(k)+1 \end{array}$$

Otherwise, *D_i_*(*j*) is assigned as a new cluster and is added to the stacks as follows:

(12) $$L=L+1\;\text{and}\;\{\begin{array}{l} {\text{Dtmp}}_{i}(L)=D_{i}(j)\\ {\text{cnt}}_{i}(L)=1 \end{array}$$

*L* refers to the maximum size of the *Dtmp* stack and *cnt_i_*(*k*) expresses how many times *Dtmp_i_*(*k*) is selected. We set *th_D* as 1 in this experiment. There is a trade-off between processing time and accuracy of the result. If we set *th*_*D* to be a large value, the processing time will decrease but the accuracy of the result will get worse. Otherwise, if we set *th*_*D* as a small value, the processing time will increase, but the accuracy of the result will improve. However, the *th*_*D* value does not matter so much.

We found two maximum values of *cnt_i_*(*k*) and their addresses *k* after obtaining the stack result at all points. If we applied the two addresses to *Dtmp*, we can find two distance values between the origin of the coordinates and two edges having the same slope. Therefore, if we obtain two distances in each case of *i* = *w* and *i* = *h*, we can finally obtain a total of four distances; *Rw*1, *Rw*2, *Rh*1, and *Rh*2. We can calculate the four corner points by using simultaneous equations between two line equations:

(13) $$\begin{array}{c} M=\left[\begin{array}{cc} \cos(\text{Angle}) & \sin(\text{Angle})\\ \cos(\text{Angle}-\pi ) & \sin(\text{Angle}-\pi ) \end{array}\right]\\ \left[\begin{array}{cccc} P1_{x} & P2_{x} & P3_{x} & P4_{x}\\ P1_{y} & P2_{y} & P3_{y} & P4_{y} \end{array}\right]=M^{-1}\times \left[\begin{array}{cccc} \text{Rw}1 & \text{Rw}2 & \text{Rw}1 & \text{Rw}2\\ \text{Rh}1 & \text{Rh}1 & \text{Rh}2 & \text{Rh}2 \end{array}\right] \end{array}$$

**Figure 10.**
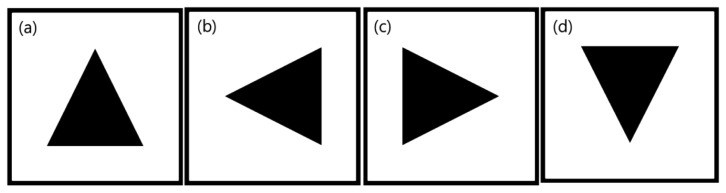
Four signs in a maker: (**a**) go straight, (**b**) turn left, (**c**) turn right, (**d**) stop.

We can check whether the contour is a real marker or not by comparing the four distances. |*Rw*1 – *Rw*2| and |*Rh*1 – *Rh*2| should have similar values with real marker length within the *th*_*D* error distance. The points, *P*1, *P*2, *P*3, and *P*4 should be integers, because the image pixel address is also integer. Therefore, four points are obtained using the *round* function in all results. The obtained four points are used to rotate the captured marker image and to match the marker with predefined markers. The rotation angle of a maker ranges from 0 to 89° because we limited the range in the Hough transform step. To modify the range from −45 to 44°, we added −45° to the *Angle* value. If the angle of rotation of the marker gets out of range, the marker will be matched with the other rotated sign. So, there are a total of four signs in a marker. Figure 10 shows the predefined four signs in a marker. We use the inside of a square contour to match the sign.

### 2.5. Marker Sign Recognition

We used a distance transform to recognize the sign in the marker. We model the recognition problem as a Bayesian MAP optimization:

(14) $$k^{*}=\arg\underset{k}{\max}P(k|I)$$

where *I* denotes the image observation and *k* denotes marker modes. Using Bayes Rule, Equation (14) can be decomposed into a joint likelihood *P*(*I* | *k*) and a prior *P*(*k*) as follows:

(15) $$P(k|I)=\frac{P(I|k)P(k)}{P(I)}\propto P(I|k)P(k)$$

If a pixel at the edge of a predefined *k* marker is (*x*,*y*) and the distance transform of the input image is *DT*, *d*(*k*) is defined as the image difference of the input image with a predefined *k* marker. We obtain an edge image of the marker by using a Sobel edge detector:

(16) $$d(k)=\sum_{\forall (x,y)}\text{DT}(x,y)/N_{k}$$

*N_k_* denotes the number of edge pixels of *k* marker. Low *d*(*k*) means that the predefined *k* marker has high similarity with the input image and high *d*(*k*) means that the *k* marker has low similarity with the input image. We obtained *P*(*I* | *k*) by applying a Gaussian model to *d*(*k*):

(17) $$P(I|k)=\frac{1}{\sqrt{2\pi }\sigma }e^{-\frac{1}{2}{\left(\frac{\left(d(k)-m\right)}{\sigma }\right)}^{2}}$$

We set *σ* to 1 and *m* to 0, because the variance in the matched images is around 1 and if *d*(*k*) is 0, it means that perfect matching exists with the input image.

When predefined markers are matched to an input image, if the probabilities of some markers have similar values, a marker having more the number of pixels at the edge should be selected. For example, as marker signs, the shapes of the characters E and F have many similar parts. If E and F have a similar probability of *P*(*I* | *K*), then character E should be selected because E has more pixels at the edge than F. In this case, E is more similar to the input image than F. Therefore, *P*(*k*) should be obtained by normalizing the inverse of the edge pixel number of the predefined markers:

(18) $$P(k)=N_{k}/\sum_{\forall k}N_{k}$$

### 2.6. Robot Simulation Test

**Figure 11.**
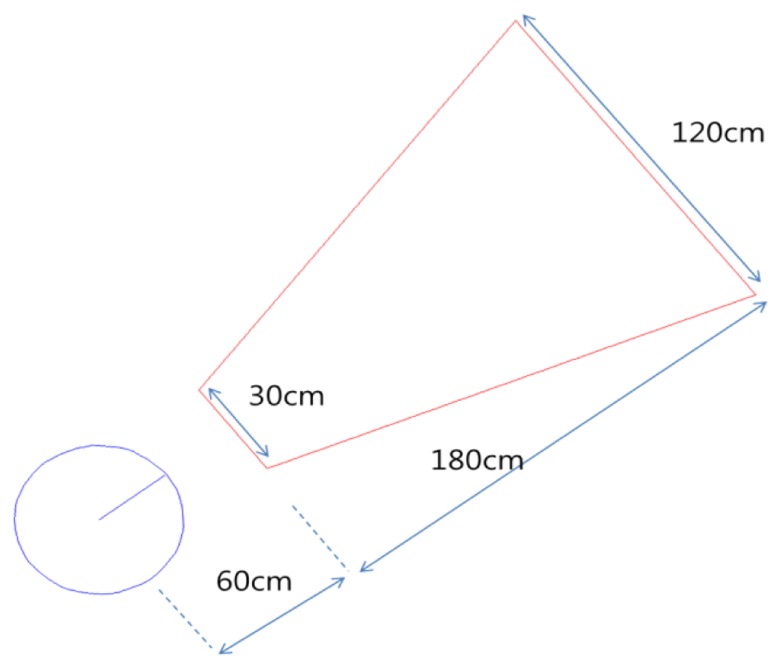
Robot model in simulation.

To confirm the performance of the algorithm, we tested the developed method in the virtual environment before applying it to real environments. We modeled the virtual environment to be as similar as possible to the real environment. Figure 11 shows a robot model. In the figure, the space of the red trapezoid denotes the sight of the camera. Each length of the trapezoid was within the recognition range of the camera in the real environment. The maximum distance for recognition is about 180 cm and the range of sight at the maximum distance is about 120 cm. The range of sight at the nearest distance was about 30 cm. Gaussian errors applied to the rotation of the robot and the rotation angle of the marker. The Gaussian error has zero mean and one standard deviation. We controlled the robot by using wheel velocity and added Gaussian errors to the wheel velocity to express skid errors on the load.

**Figure 12.**
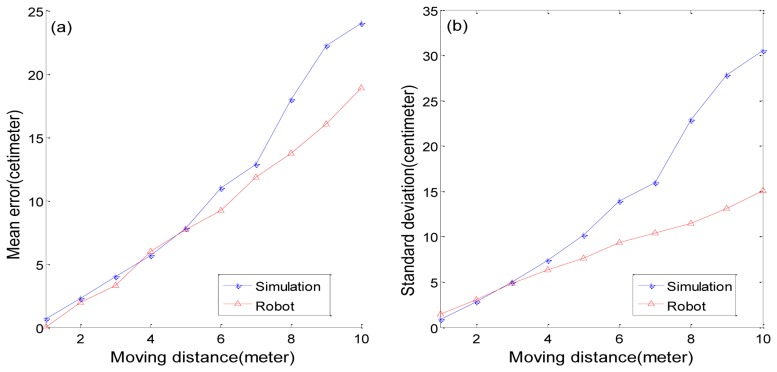
Movement test results in real environment and in virtual environment. (**a**) mean distance errors in moving distance (**b**) standard deviations in moving distance.

Figure 12 shows the movement results of the robot in the virtual environment and in the real environment. In Figure 12a, the *x* axis refers to the movement distance and the *y* axis refers to the mean of distance errors. Figure 12b shows standard deviations. The virtual robot has a larger standard deviation than the real robot, but that is reasonable because we can't apply every possible problem to the simulation. We need to leave some possible errors to avoid sudden problems in a real environment.

**Figure 13.**
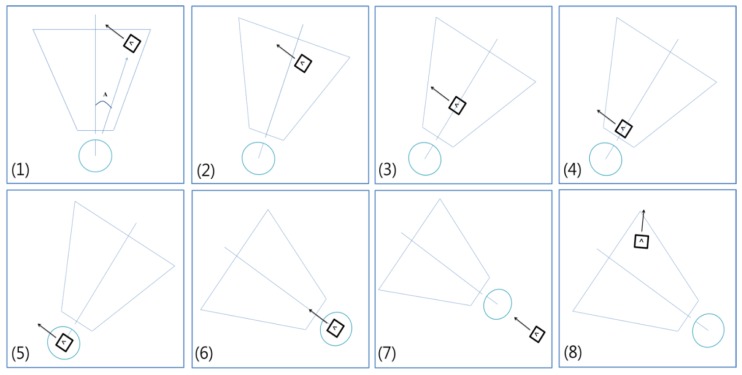
An example of robot guidance process.

Figure 13 shows the robot guidance process into ten steps. First, if the robot finds a marker, it calculates the location of the marker. If a marker is detected, the robot can follow the marker wherever it is. Steps from 2 to 4 mean that the robot is approaching to the marker. To exactly follow the marker, the robot should reduce the moving speed as the distance between marker and robot is decreased. Step 5 shows the robot finally reaches to the marker and it turns the direction as the marker direction in step 6. In steps 7 and 8, the robot moving with the marker direction and find other marker.

**Figure 14.**
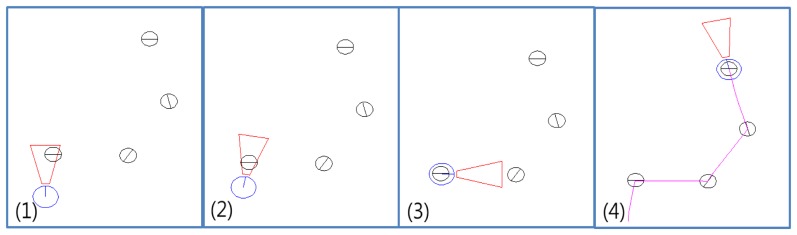
Robot guidance process test.

The initial orientation of the robot does not need to point at the sign go straight. By using four direction signs in the Figure 10, it is possible to express all direction range from 0° to 360°, because each sign has range 90 degree around each direction. Therefore, if a marker is in the range of detection, the robot can follow the marker. In the Figure 14, the numbers in the figure means the order of robot guidance process. In the initial stage, the robot starts with detecting a marker. Next, the robot move following the marker. Figure 14-4 shows a trajectory of the robot. We can confirm that if a marker is in the range of detection, the robot can follow the marker.

## 3. Experimental Result

### 3.1. Object Occlusion Test Result

**Figure 15.**
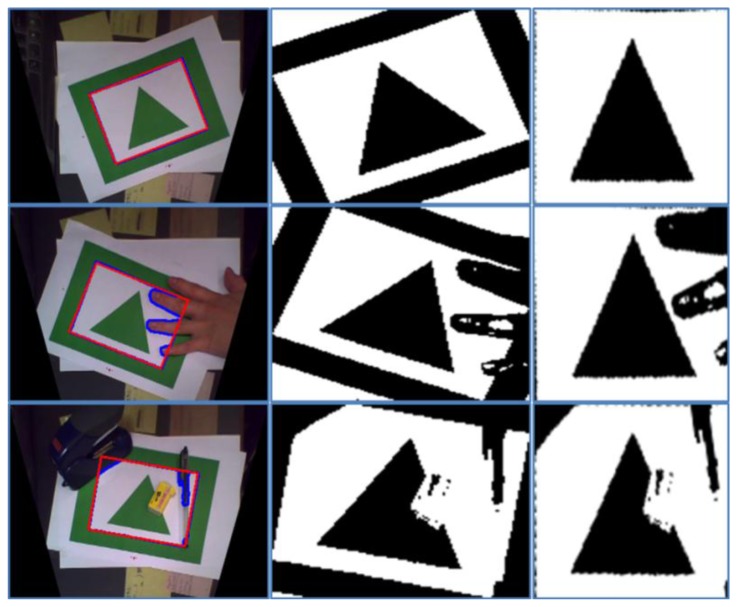
Examples of overlap in marker recognition.

By using a Hough transform, we can optimize overlap problems. In the experiment, we match the square shape with candidates by using the Hough transform because we assume that the marker should be square. This approach can reduce the influence of image occlusion. Figure 15 shows some examples of image occlusion. The first column shows the bird's eye view image with marker recognition. The blue line express contour of the candidate and the red line describes the estimated marker shape using the proposed method. The second column shows the binary image of the candidate. The third column shows detected and rotated marker image. We can confirm that although some occlusions occur in the image, the proposed method exactly extracts the marker. These results show that the proposed method effectively overcomes overlap problems.

### 3.2. Test Result of Rotation Angle of Marker

**Figure 16.**
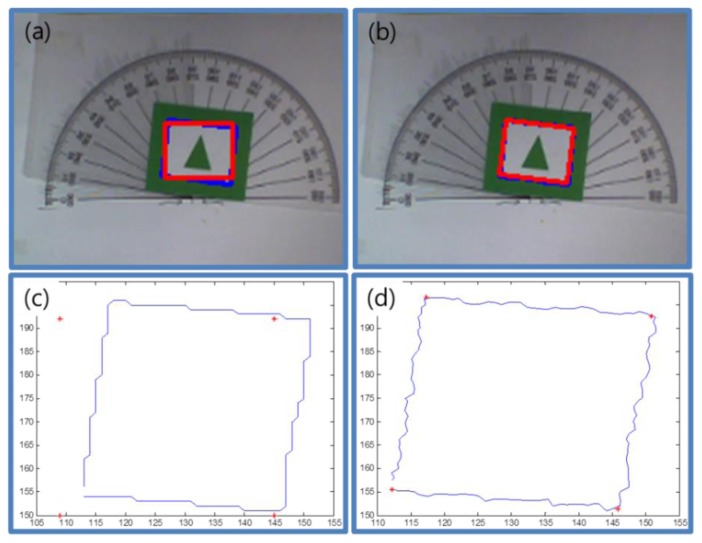
Comparison between normal state and with added noise. (**a**) Normal Hough transform results. (**b**) Adding noise to Hough transform results. (**c**) Blue line refers to normal contour points of a marker and red points indicate estimated corner points of the marker. (**d**) Blue line refers to adding the noise contour points of a marker and red points indicate the estimated corner points of the marker.

We used the Hough transform to estimate the rotation angle of the marker, but the Hough transform could not find correct edge lines if the edge of the contour has a step shape. To avoid this problem, we added some noise to the contour. We can make the edges smooth by adding some noise to the contour and obtain more exact results by applying the Hough transform to the smoothed edges. Figure 16 shows an example result. Figure 16a shows the result of the Hough transform with less noise. The red square denotes the detected marker shape and the blue square denotes the contour of the original marker. The marker is detected, but the rotation angle of the marker is not correct.

Figure 16c shows the contour points (blue) and recognized corner points (red). The contour points form the shape of the step. This shape can cause some errors in estimating the rotation angle of the marker. Figure 16b shows a result of marker detection due to adding some noise to the marker contour. The estimated square shape (red) is very similar to the contour of the marker (blue). This means that the rotation angle of the marker is correctly estimated. In Figure 16d, we can confirm that the edges are smoothed and the estimated four corner points are nearly correct.

**Figure 17.**
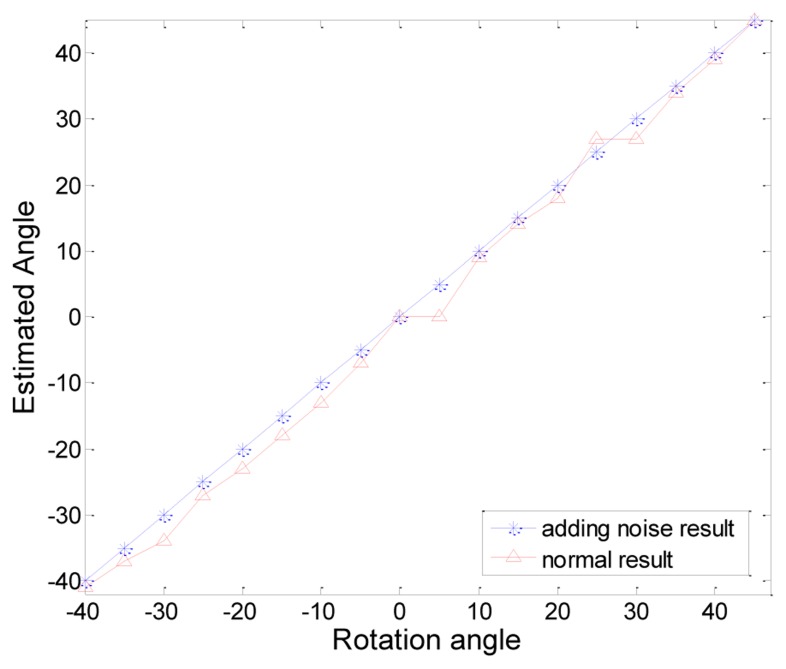
Comparison performance results between normal estimation and adding noise result.

Figure 17 shows the performance of the rotation angle estimation when measured closest to the camera. We tested the rotation angle at intervals of 5 degrees from −40 to 45 degrees. In the figure, the blue line shows the result of adding some noise to the contour and the red line shows the result of the original contour. We obtained almost accurate results by smoothing the edges with some noise. There was no any rotation error near the camera, so the blue line was linear. However, the non-adding noise method caused about 2 degrees of error on average. If the noise is too large, the error in the rotation angle increases. But if the noise is too small, the step shape has not disappeared. Proper values need to be selected. In this experiment, we applied random noise value between −0.006 and 0.006 to the contour.

**Figure 18.**
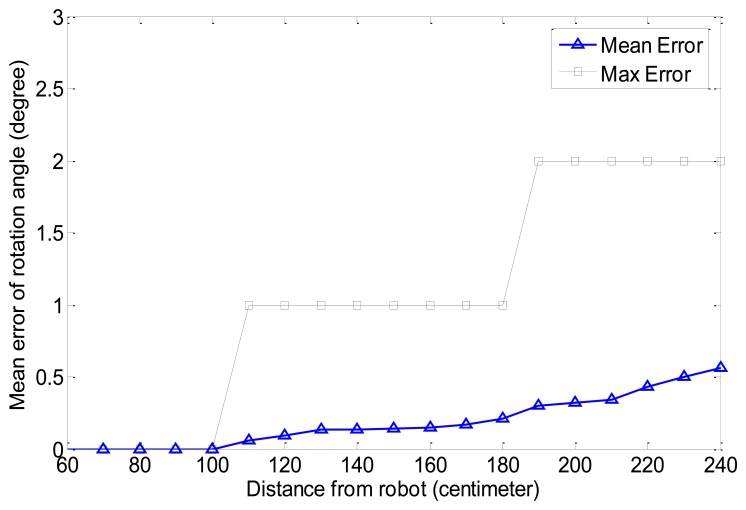
Error in rotation angle of marker according to distance from robot.

We tested the error in the rotation angle of the marker according to the distance from the robot. Figure 18 shows the result. The black line shows the max error in the rotation angle according to the distance. As the distance increases, the max error is also increasing but the max rotation error is below 2° at the max distance. The blue line shows the mean error in the rotation angle. The mean error is less than 1° at the max distance. When we tested the rotation error, we could obtain accurate results within less than 100 cm. The max error was below 1 within 180 cm and the mean error was also almost 0. Therefore, we can obtain nearly correct angle result within 180 cm. As the distance increases, the error is also increased. The image is expended and changed as distance from the camera increases in the image when the image is transformed to bird's eye view. However, although the rotation error is at a maximum for the farthest distance, the error does not have any bad effect on the motion of the robot. The max mean error at the farthest distance is less than 1 and the rotation angle for the farthest distance has no mean because the robot checks the rotation angle of the marker at the closest distance. The proposed method is almost accurate for estimating the rotation angle of the marker. This method is appropriate for applying vision-based robot control. However, camera calibration is very important to obtain accurate results with the proposed method. If there is some camera distortion, we can't obtain accurate results and the errors will increase.

**Figure 19.**
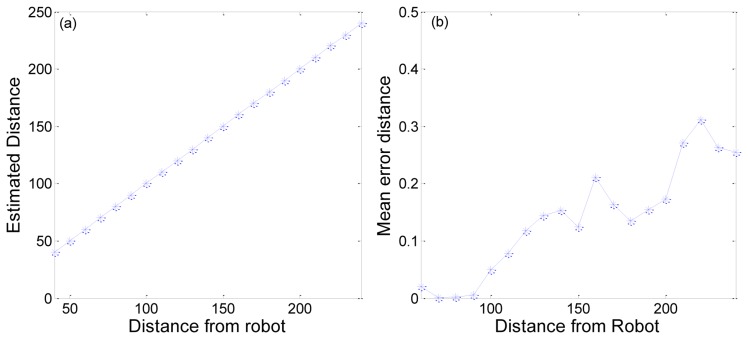
Estimated distance and mean error distance.

We also tested the location of a marker from the bird's eye view image. Figure 19a shows a comparison between estimated distance and real distance. We can obtain a mostly accurate distance. The mean error in distance is about 0.1382 cm. This error is reasonable, because the image pixel is discrete and the method use the distance per pixel. Figure 19b shows the mean errors at each distance from the robot. Even if the error value increases with the distance from the robot, the mean distance error is less than 0.3 cm. This means that we can obtain an almost accurate location for the marker.

### 3.3. Marker Recognition Result

**Figure 20.**
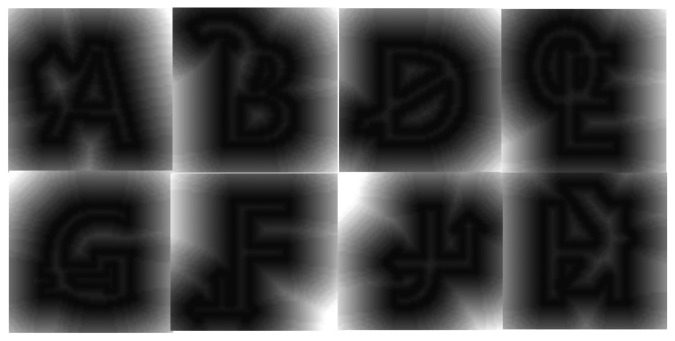
Examples of distance transform of markers with occlusions.

We use the distance transform method to obtain the probability of matching between the detected marker and a predefined marker. Figure 20 shows some examples of distance transformation for markers with occlusions. We compared the edge of predefined markers with the distance transform image of the detected marker. We chose the highest probability of *P*(*k* | *I*) in Equation (15) as a recognized marker sign. We used about 600 images to test. Each image was normalized to 64 × 64 pixels to compare the image. When we tested the recognition rate of the sign in the marker with various images such as occlusion, partially deleted and tilted images, we could obtain average 98.87% accuracy.

### 3.4. Robot Movement Test Result

**Figure 21.**
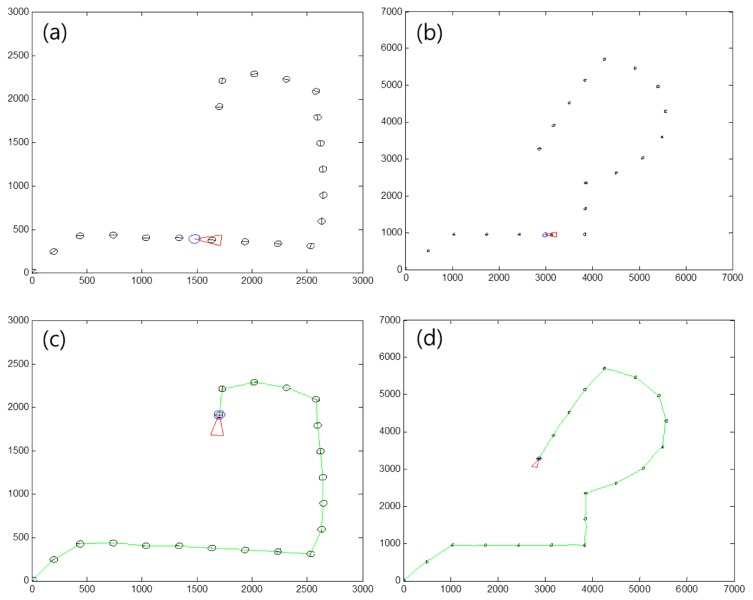
Robot simulation examples. (**a**) distance between markers is 300 cm. (**b**) distance between markers is 700 cm. (**c**), (**d**) final results of robot movement at each marker distance. The green line shows the moving path of the robot.

To find a proper distance between markers, we tested the robot simulation in a virtual environment. Figure 21 shows examples of a marker map and the moving path of the robot. Figure 21a shows a marker map with a distance of 300 cm between markers. Figure 21b shows a marker map having a distance of 700 cm between markers. The size of space Figure 21b is larger than that in Figure 21a, because the distance between markers is bigger than that in Figure 21a. In Figure 21c,d, the green line refers to the movement of the robot. This shows the robot can follow the markers without any problems. We made five kinds of marker routes and we tested the robot movement 30 times at each map for changing the distance between markers from 100 to 2,000 cm.

**Figure 22.**
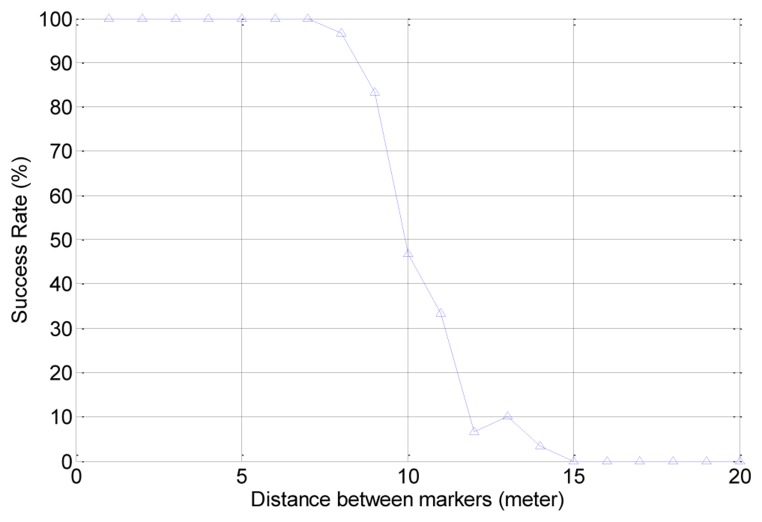
Success rate as a function of distance between markers.

Figure 22 shows the simulation results. In the test, if the distance between the markers is less than 700 cm, the robot can perfectly follow the route. However, as the distance between markers increases, the success rate sharply decreases. This means that the robot loses its path and gets out of the route if the distance between the markers is too large.

**Figure 23.**
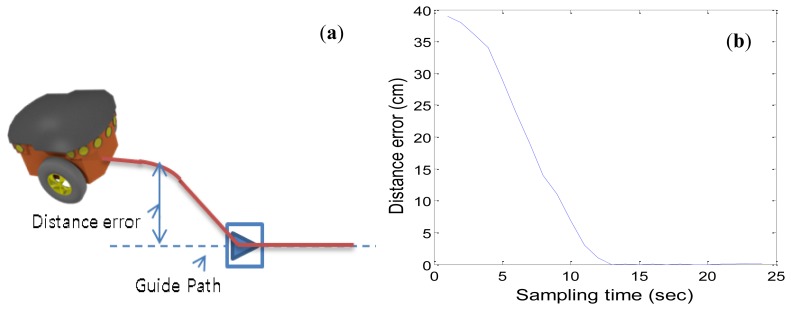
Path following test using a Pioneer3DX.

Figure 23 shows a path following test using a Pioneer3DX. Figure 23a shows a situation that the robot has some distance error from the guide path. This situation is possible and can be usually happened, because the robot is only depending on a marker for path following.

Figure 23b shows a result of correcting its path after that the robot finds a marker. Even if there are some route errors, the robot can correct its route by following a detected marker. After the simulation, we tested the robot following in the hallway. We selected the distance between markers to be 700 cm according to the simulation result. When we tested the robot several times in the real environment, we could confirm that the proposed method works well and the robot did not leave the path. From the test result, we can confirm that the proposed method can be used in an indoor environment. In our environment, there was no significant slip of the robot. If there are some significant slips, the algorithm can't work well over long distances between markers. However, this problem may be solved if some compensation method is applied to the robot for reducing moving errors.

## 4. Conclusions

We have proposed a new approach for an AGV system. An AGV system based on marker recognition has some advantages in terms of the cost of installation and changing routes. To control the robot accurately, we developed methods to obtain an accurate rotation angle for the marker and to calculate an accurate distance from the robot. We used hue and saturation information to obtain a robust candidate image. A bird's eye view image was used to obtain an accurate location and position for the marker. We also applied a Hough transform method to robustly recognize the marker and to obtain an accurate rotation angle for the marker. To recognize the sign on the marker, we used a probabilistic approach by using distance transform matching. A feature having high probability is selected as a captured marker sign. Although there are some occlusion or tilt errors, the average recognition rates are 98.87%. We also tested the robot simulation to find the proper distance between markers. By testing the robot in simulation, we could find the maximum possible distance between markers. From the simulation result, we could confirm that if the distance between markers was less than 7 m, then the proposed method worked well. We also proved the simulation results by testing the robot in a real environment. We used a computer system with a 2.6 GHz CPU and 2 GB RAM memory. We used Matlab(r2010a) for simple algorithm tests and we used C language with openCV for the on-board robot programs. Total processing time was about 78 ms for every 640 × 480 frame. The proposed method can work in real time.

## Figures and Tables

**Table 1.** Recognition rates of signs A–J.

**A**	**B**	**C**	**D**	**E**
98.8%	98.7%	100%	100%	97%
**F**	**G**	**H**	**I**	**J**
96.6%	97.4%	99.2%	98.8%	98.2%
**Forward**	**Stop**	**Left**	**Right**	**Average**
99.5%	100%	100%	100%	98.87%
